# Is There a Degeneration in the Teeth? If so, to What Is It Attributable?

**Published:** 1855-10

**Authors:** Wm. A. Pease


### ARTICLE XI.

### *Is there a Degeneration in the Teeth? If so, to what is it Attributable?* By Wm. A. Pease.

Twenty-five or thirty years since, dentistry was, comparatively, in its infancy. None of the improvements we now possess had been made, or if they existed in a crude shape, they were such ghostly simulacra that none but the owl-eyed could discover in them the lineaments of their descendants. From that time to the present the improvements have been so rapid, they have followed so closely upon the heels of one another, we have been filled with a constant surprise, and have found little leisure to examine them and see to what good they conduce, or whither they go.

It will fall within the scope of this paper to examine the condition of the human mouth then, as compared with it at present; to note the several successive changes that have occurred, whether induced by natural or artificial causes; trace them to their sources, and form an estimate of their present and prospective importance, not only as effecting the condition of the mouth, and exerting considerable influence upon it; the habits and physical health of society will be glanced at in different eras, to determine what effect, if any, they exert upon the teeth, or have exerted upon them within the authentic period of dental history. And art will be subjected to a slight inquisition to discover, notwithstanding its pretensions, if society derives from its labors a benefit commensurate with its expectations and the solid dollars it pays for them.

In the absence of dental data and dental records that afford us direct and positive light, we will have to resort to the indirect and unwritten, which, under certain circumstances, are held to be as conclusive and authoritative as the written, and, like the common law, are quoted and respected. Thus, we have no reports founded on the careful collection and digestion of a large number of cases, showing the time that will elapse before decay will appear in a tooth after a set of artificial teeth has been attached to it. Yet few dentists of much experience and observation will doubt that decay will commence, in a majority of cases, within a few years; and in a young subject, though it may be partially arrested by art, will ultimately destroy the tooth.

In taking twenty-five years as the initial of our retrospect, we will not have passed beyond the period of persons now living, who were then on the stage of life and occupying a similar position with their children, now in the meridian of life, whose mouths we wish to compare together; and we will not be deprived of their auto-dental history, and the means they enjoyed, or had recourse to, to preserve their teeth (if any) as compared with what are now employed.

In so short a period there can be no palpable contrast, but much of that sameness and those general features will be observed, both in lineaments and constitution, that hereditacy produces—the children will represent the sire, and this generation the preceding; and it is precisely for this reason we examine them, to see how closely the resemblance holds good, and if there is a difference, to discover in what it consists, and what are its distinguishing characteristics. Whether, as art and science advance, the teeth partake of the improvement—are stronger, healthier and more enduring, from the increased means for insuring their health which science affords; or, in despite of the aid of science, there is a visible deterioration—a tendency to contract disease, with less power of resisting it. We have frequently examined the teeth of father and son, mother and daughter, and occasionally we have had three or four generations together, and have taken models to institute a more minute examination at our leisure. In these examinations the hereditary tendency has been observed, and the peculiarities that sometimes overleap one generation to find in the cross of the third, the peculiarity of temperament favorable to their development. At times we have seen the same peculiarities descend continuously in the same family through several generations, as is generally the case, where the family lineaments are preserved. But there was this difference, the physical health and power of resisting disease in the teeth was no where observed to descend with the family configuration and expression. The teeth now seem to be more porous, and to be permeated by a greater number of blood-vessels and nerve filaments; and we have failed to observe, as is generally inculcated, that these porous teeth eventually become dense. That they increase in density, we are prepared to admit; that their minuter cells or canaliculi are sometimes obliterated is equally obvious; but that any length of years, or course of diet, will produce that density of structure which characterizes their ancestors' teeth, is improbable. There appears to be an exaltation of the vascular and nervous dental system that is not hereditary but acquired, which seems under certain circumstances to increase, subject to what limits, if limitable, we are ignorant.

If we recur to New England, where families can be more easily and certainly traced; where some of the descendants of the first settlers still remain near the original homestead; where the family history is cherished and better preserved, and the peculiarities of the ancestry (if unwritten) are handed down from generation to generation, I think we can establish the following points, viz. 1st. In a given population now and twenty-five years since, between the ages of 20 and 30, there will be found a greater number who have lost teeth now than then. 2d. Disease commences earlier and runs a more rapid and destructive course, notwithstanding the greater care bestowed upon the teeth now than then.

Twenty-five years since in the rural districts of New England a dentist was scarcely known, and medical skill had not obtained that celebrity it now enjoys. The physician, it is true, was well read, and a man of parts, but he had not received that discipline, experience and familiarity with disease a modern practitioner can acquire at a modern college. He knew little about the teeth, and attached importance to them only as a grinding apparatus, that occasionally vexed him by requiring a perplexing operation. He studied not their diseases, nor deemed them worthy of study—employed few palliatives, and believed when once diseased there was no radical cure but a radical removal of them from the jaw. Plugging was scarcely known and seldom attempted; the mineral and actual cauteries silenced tooth-ache where narcotism failed. If his medicines were few, his instruments were fewer, and so primitive in construction that only the most favorable teeth could be extracted. The value of the teeth was not taught, because the loss of them was comparatively unfrequent; cleanliness was not inculcated, a reckless use of the teeth for improper purposes was common, and disease once established was unmolested till terminated by the key or the slow progress of decay. Decay was synonymous with the ultimate loss of the tooth, and the loss was an irremediable evil. An artificial tooth was unheard of, or, if heard of, was treated as a myth, as unreal and unsubstantial as the fabulous creatures in the Arabian Nights. If Washington and some other historical persons were known to have worn artificial teeth, they were regarded more as an appendage of state—a part of the paraphernalia of office—more useful as a show than as bread and butter eating machines; such was the condition of dentistry then, and such the condition of the teeth, the nature of their treatment, and their liability to disease. Now, when every hamlet sustains a dentist, and the cities are swarming with them—when the attention of the community is aroused to the importance of healthy teeth, reasoning, a priori, we must conclude that, cæteris paribus, the teeth are seldom lost; that though disease does occasionally attack them, it is soon arrested, its contagious and contaminating effects are removed, and the integrity of the mouth is preserved by the superiority of modern dental skill. Is this true?

The better to institute a direct comparison to determine whether the young now starting in life possess as good physical health and teeth as their early ancestors—whether the lamentable loss of the teeth now so generally witnessed, is the result of a want of skill in dental practitioners, or arises from causes beyond remedial control, we will take that patriarch, the venerable Maj. H——, as a type of the physical and dental health of the earlier inhabitants of this country. He shouldered his musket and followed several older brothers to the army, and helped to win his country's independence, and still lives, a hale old man, surrounded by a numerous progeny, to enjoy the fruits of his early toil. He has recently lost the partner of his youth, manhood and old age. We do not select him as an isolated case, but because we are familiar with his history, have has frequent occasion to operate on the teeth of some of his descendants, and have examined them with that care that enables us to speak with confidence of their condition.

Other similar cases might be easily adduced, but this one will suffice. Maj. H.—— was but little above the medium size, strong, healthy and firmly knit—of a sanguine nervous temperament.

His teeth, never white, were of a clear yellowish cast, of a dense structure, and large, for a man of his size. His wife, his equal in years and health, was his superior in fullness of person, though possessed of no extra flesh; her temperament was a nervous bilious, and her teeth were as healthy as his own; and within a few years we have extracted some of them, free from caries, but loose from alveolar irritation. If they are credible witnesses, their teeth, when young, were no better than the generality of their neighbors—several of whom we have personally known, among whom tooth-ache was not a common occurrence, and the loss of a tooth was unfrequent.

If we assume, then, that theirs was a fair type of the teeth one hundred years since, as I think we safely can, we will have a starting point, or standard, to which to refer in our subsequent investigations and observations. That their children should possess their characteristics, physically and dentally, will be expected, which they do to a certain extent. The oldest children inherit the father's temperament and physical and dental peculiarities, while the younger more nearly resemble the mother. All of them have lost some teeth, generally unnecessarily, and two sisters have worn partial superior sets since fifty years of age. The one principally from tartar accumulations and alveolar irritation; the other's teeth perfectly antagonized and the incisors became worn away to the gum.

In the children in the next descent, the change in the dental development is more marked; the size of the teeth is not as great and there is a plain diminution in the quantity of the inorganic matter they contain; and, though still large, strong and healthy, they are more obnoxious to the attacks of caries, and require the aid of the dentist to plug them before they arrive at the meridian of life; indeed, a son and daughter early wore pivot teeth.

Now, it cannot be objected that the children of Maj. H—— married into a less vigorous and healthy stock, and ergo, their children's teeth are impoverished and lack the inorganic matter to interpose a successful barrier to the attacks of disease; for, there was an equality of dental and physical health in the various families with which they intermarried. Their health and teeth are not above the average of the community in which they reside, while their habits are nearly the same, and their children are subject to the same educational, social and climate influences as their neighbors, and to the same general hygienic and operative treatment.

We now come to the last generation—the great grand children of Maj. H——. They are not as yet numerous, though some of them have their full denture, and consequently display all the physical and dental characteristics necessary to this inquiry. Again, it is proper to remark, they have been within the pale of the same influences as others of their age, have suffered from like causes, received no better attention, and present a fair average of dental health. If their teeth are slightly above the average in size, there is not sufficient difference to constitute a noticeable feature, nor is the density of their structure, or the perfection of their enamel particularly in their favor; and there seems to be a predisposition to caries similar to that observed in others of their age, which, if unmolested, would doubtless run a rapid and destructive course.

After this rapid glance at these several generations, we will take a review of them, in order to keep some of the more prominent points in sight—to determine how far hereditacy extends and leaves traces of the strong original stock, and how far other equally strong stocks have influenced it, by modifying or obliterating it, or engrafting upon it their own peculiarities.

The two oldest children, a daughter and son, inherit the father's health and temperament, while five younger, two daughters and three sons, more nearly resemble the mother.

The oldest daughter is unmarried. She has suffered little from caries, though within ten years she has lost several teeth from tartar. The oldest son has still very healthy teeth, though he has lately lost a first superior molar from absorption of the socket; the inferior antagonizing tooth, having been extracted while young. His teeth resemble his father's. His wife, though possessed of vigorous health, has lost most of her molar teeth, and now wears four pivot teeth—the other members of her family are healthy and have better teeth than she has. The children of this pair exhibit a feature we wish to note. The oldest, a daughter, reproduces the paternal grandmother in features and physique, though a little more slender, and her teeth present all of her characteristics in arrangement, color and structure, though considerably less in size. She has suffered little from caries; her teeth are healthy, but there is a strong proclivity to alveolar irritation, which is easily aroused by any exciting cause. The second daughter does not so much resemble the paternal side. She has suffered considerably from caries, her bicuspid and molar teeth were plugged while young, several of which have since crumbled away. They were soft and friable. Her mouth is little inclined to tartar accumulations, or alveolar irritation. The third child, also a daughter, again, very nearly resembles the paternal grandmother. Her teeth are large, regular and particularly strong and healthy, though when quite young she suffered slight attacks of caries, which were easily managed. The alveolar irritation of her grandmother, in her mouth, very nearly disappears.

Here this branch terminates, and we will return to the second daughter of Maj. H——, who we have already observed wears artificial teeth. That her teeth were originally healthy is evidenced by the fact, that she has worn a plate attached to the first or second superior molar for near ten years, without visible injury to them. Like her mother, she has lost several teeth from alveolar absorption, and she has also suffered some from caries.

She was married into a healthy family were the sanguine temperament predominated. Her husband was about her own age. He was six feet in height, and otherwise well proportioned. Blessed with vigorous general and dental health, he never employed a dentist, though some of his molar teeth were extracted by a physician, and the incisors were worn away nearly to the gum. They have several children, some of whom have children whose denture we wish to examine.

In speaking of her descendants, it is proper to remark, that as she resembles her mother, so do also her children, though not so much as the daughters of the oldest son; but in her family the tendency to irritation of the dental socket nearly disappears. Her children have fair average teeth in size, health and density in structure. They are indebted to dentistry for the preservation of them, and a second son and daughter for the insertion of two pivot teeth.

Her first daughter married into a healthy family. She has a daughter, whose denture is complete. As she is the first one in the third generation, we shall examine her teeth with care.

Her father, Mr. L——, is a healthy, middle aged man, not above medinm height, broad and full chested. Originally he had healthy teeth. From neglect and bad dentistry, he now wears a front artificial set, and he has lost several of his molars and bicuspids. The daughter has remarkably fine, regular and large teeth, which in some respects resemble the teeth of both parents, but more nearly the mother's. They early began to decay, and it is probable, notwithstanding great care is bestowed upon them, she will be unable to retain all of them long on account of their soft consistence.

It will suffice to remark, the three youngest sons of Maj. H. still retain most of their teeth, notwithstanding they have uniformly neglected them; and they are but slightly indebted to dental skill. Each has a son, who has children, but their denture is not sufficiently advanced to determine its character.

It is no part of our purpose in selecting Maj. H——, as a standard to present an isolated or extreme case. We seek a fair representative of New England people—a type of the age. We do not think he was endowed with a greater measure of health than his early and manly associates, though from accident and disease, they have one by one disappeared; often after passing the threescore and ten years. The heads of the families, whose children united with his, though none of them attained quite so great an age, were nevertheless venerable and long lived, and their children show no visible indication why they may not follow long in their course. None of them, or their children, have derived but little if any benefit from dentistry, and their children's children, now between twenty and forty years of age, have not contributed much to dental support; but *their* children now bid fair to present to the profession a liberal honorarium.

The review of the denture of these four generations may be concluded under the following heads:

1. There is a difference in the expression of the children's teeth now, as compared to their early ancestors.

2. There is a difference in the constitution of the teeth; the fourth generation possess less of the inorganic matter than their early sires.

3. There is a difference in the size of the teeth, and the regularity and fullness of the denture.

4. There is a difference in the period and manner of carial attack, its type and the rapidity of its course.

And finally, we think there is a difference in the resistance they offer to luxation.

In discussing the first proposition it is proper to remark, to discover a difference in expression in so small and apparently so unimportant a thing as a tooth, and to divest it of the modifying influence of the features and lips, requires an æsthetical education, and that delicacy of observation such an education alone can produce.

To most people a tooth is a tooth, whatever its form, size or color; and many medically learned would fail to find in its expression, any indication of its physical character, or health, or of the health of the individual. To us, there is a something in the expression of the teeth indicative of character, which we cannot better describe, than as a sensation or perception akin to that we receive from the contemplation of the features. Conjoined with the features, they are the index of the man. As they are large or small, regular or irregular, prominent or depressed, the expression of the mouth varies and is firm and manly, bold, avaricious, cowardly or puerile. An impoverished stock is not more surely indicated by the want of muscular development, than by the osseous. The first glance satisfies us of the health of the teeth as it does of the temperament of the individual; and yet, were we asked to designate the condition or indication on which we predicate our judgment, we could only answer, there is no form or tint which determines it; size, color and regularity alone are insufficient—it is intangible, but intelligible; it is experience. Perhaps the greatest difference between a naturally healthy and unhealthy tooth, when free from stains, consists in the greater transparency of the enamel of the unhealthy one, it seems thinner, more vitreous—and the body of the tooth also partakes of the delicacy, and seems more penetrable to light. There is often a bluish or purple color, sometimes mottled, and seldom a clear white or yellow as in healthy teeth. Generally they are stained, which obliterates the natural expression and gives them a dull, opaque, or chalky appearance.—*Dental News Letter.*

